# The genome sequence of a digger wasp, *Ectemnius lituratus *(Panzer,1805)

**DOI:** 10.12688/wellcomeopenres.20337.1

**Published:** 2023-12-01

**Authors:** Liam M. Crowley, University of Oxford and Wytham Woods Genome Acquisition Lab, Darwin Tree of Life Barcoding collective, Wellcome Sanger Institute Tree of Life Programme, Wellcome Sanger Institute Scientific Operations: DNA Pipelines collective, Wellcome Sanger Institute Tree of Life Core Informatics Team, Tree of Life Core Informatics collective, Darwin Tree of Life Consortium

**Affiliations:** 1 Department of Biology, University of Oxford, Oxford, England, UK

**Keywords:** Ectemnius lituratus, a digger wasp, genome sequence, chromosomal, Hymenoptera

## Abstract

We present a genome assembly from an individual female *Ectemnius lituratus* (a digger wasp; Arthropoda; Insecta; Hymenoptera; Crabronidae). The genome sequence is 235.1 megabases in span. Most of the assembly is scaffolded into 13 chromosomal pseudomolecules. The mitochondrial genome has also been assembled and is 29.67 kilobases in length. Gene annotation of this assembly on Ensembl identified 9,724 protein coding genes.

## Species taxonomy

Eukaryota; Metazoa; Eumetazoa; Bilateria; Protostomia; Ecdysozoa; Panarthropoda; Arthropoda; Mandibulata; Pancrustacea; Hexapoda; Insecta; Dicondylia; Pterygota; Neoptera; Endopterygota; Hymenoptera; Apocrita; Aculeata; Apoidea; Crabronidae; Crabroninae; Crabronini; Crabronina; *Ectemnius*; *Ectemnius lituratus* (Panzer, 1805) (NCBI:txid2495015).

## Background

*Ectemnius lituratus* is a small to medium sized digger wasp in the family Crabronidae. It is widespread across much of northern Europe, and in the UK it is common across southern England. It is black with yellow markings on the scapes, pronotum, scutellum, tibiae, tarsi and tergites. The clypeus is covered with shining silver hairs, the mesonotum is longitudinally striate, and there is a poorly-developed tooth midway along the inner margin of the mandibles. Unusually for aculeates, the male *Ectemnius* do not have an additional antennal segment, with both sexes possessing 12 segments. Male *E. lituratus* are one of two British species with simple untoothed antennae.

*E. lituratus* occurs in a range of habitats, especially woodlands and forested areas. It is univoltine, with a later flight period than other *Ectemnius* species, from June to September. Females hunt medium-sized Diptera, typically calypterates including Anthomyiidae and Cordyluridae ( Lomholdt, 1975). Nests are constructed in cavities in dead wood, including dead trees, stumps and fence posts. Adults are strongly associated with the flowers of umbellifers, especially wild parsnip, cow-parsley, hogweed, wild angelica and greater burnet-saxifrage, on which they can be abundant ( Falk, 1998).

The complete genome sequence for this species will facilitate studies into the evolution of hunting strategies, reproductive systems and Hymenopteran taxonomy.

## Genome sequence report

**Figure 1.**
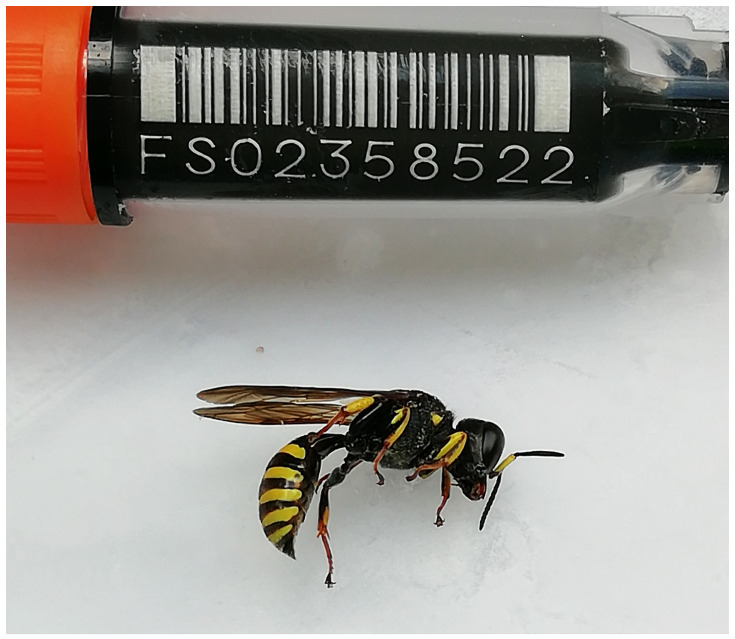
Photograph of the *Ectemnius lituratus* (iyEctLitu1) specimen used for genome sequencing.

The genome was sequenced from one female *Ectemnius lituratus* ( Figure 1) collected from Wytham Woods, Oxfordshire, UK (51.77, –1.34). A total of 110-fold coverage in Pacific Biosciences single-molecule HiFi long reads and 144-fold coverage in 10X Genomics read clouds were generated. Primary assembly contigs were scaffolded with chromosome conformation Hi-C data. Manual assembly curation corrected 196 missing joins or mis-joins and removed one haplotypic duplication, reducing the scaffold number by 44.17%, and increasing the scaffold N50 by 143.70%.

**Figure 2.**
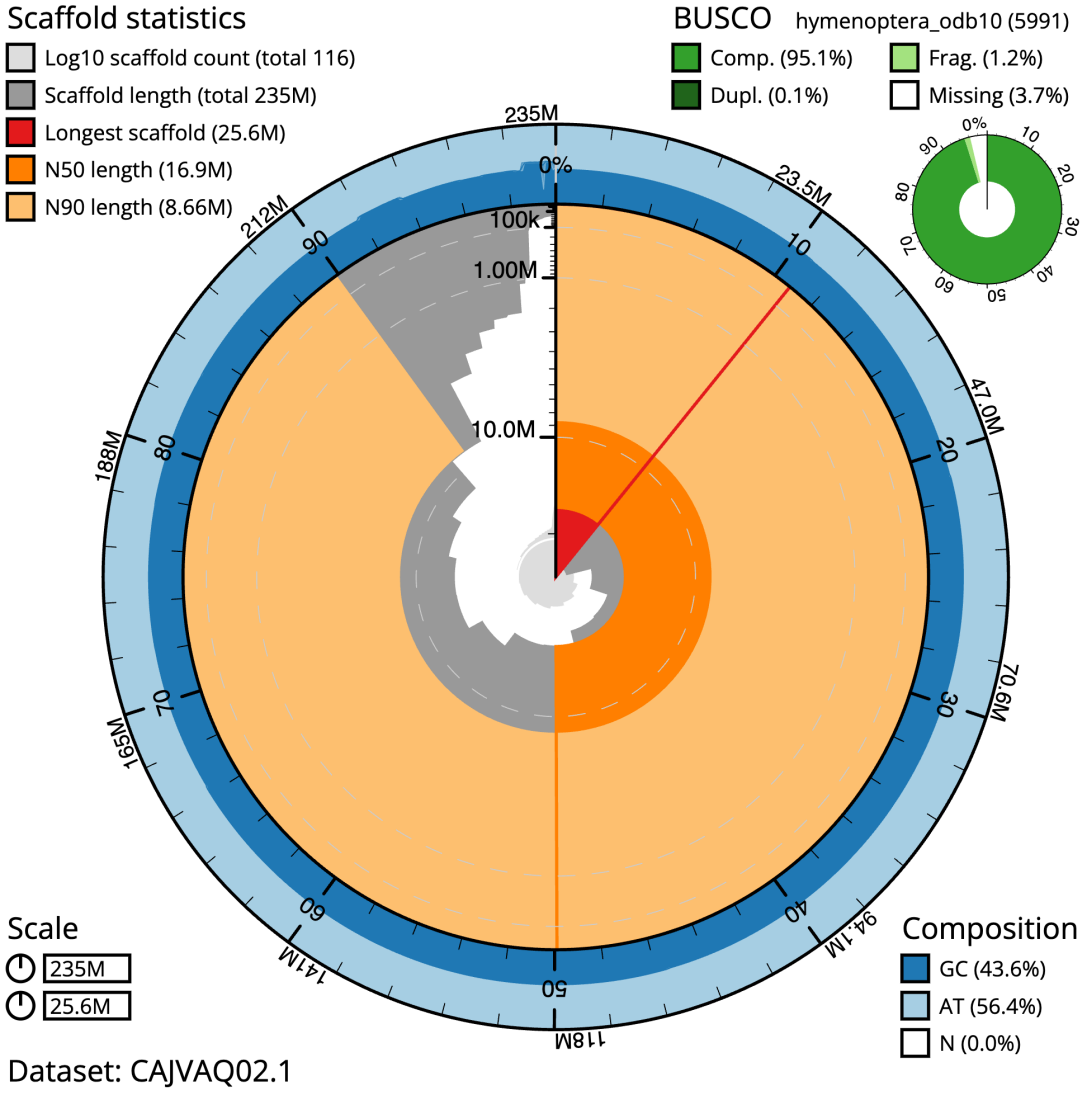
Genome assembly of *Ectemnius lituratus*, iyEctLitu1.2: metrics. The BlobToolKit Snailplot shows N50 metrics and BUSCO gene completeness. The main plot is divided into 1,000 size-ordered bins around the circumference with each bin representing 0.1% of the 235,170,256 bp assembly. The distribution of scaffold lengths is shown in dark grey with the plot radius scaled to the longest scaffold present in the assembly (25,570,032 bp, shown in red). Orange and pale-orange arcs show the N50 and N90 scaffold lengths (16,908,772 and 8,661,520 bp), respectively. The pale grey spiral shows the cumulative scaffold count on a log scale with white scale lines showing successive orders of magnitude. The blue and pale-blue area around the outside of the plot shows the distribution of GC, AT and N percentages in the same bins as the inner plot. A summary of complete, fragmented, duplicated and missing BUSCO genes in the hymenoptera_odb10 set is shown in the top right. An interactive version of this figure is available at https://blobtoolkit.genomehubs.org/view/Ectemnius%20lituratus/dataset/CAJVAQ02.1/snail.

**Figure 3.**
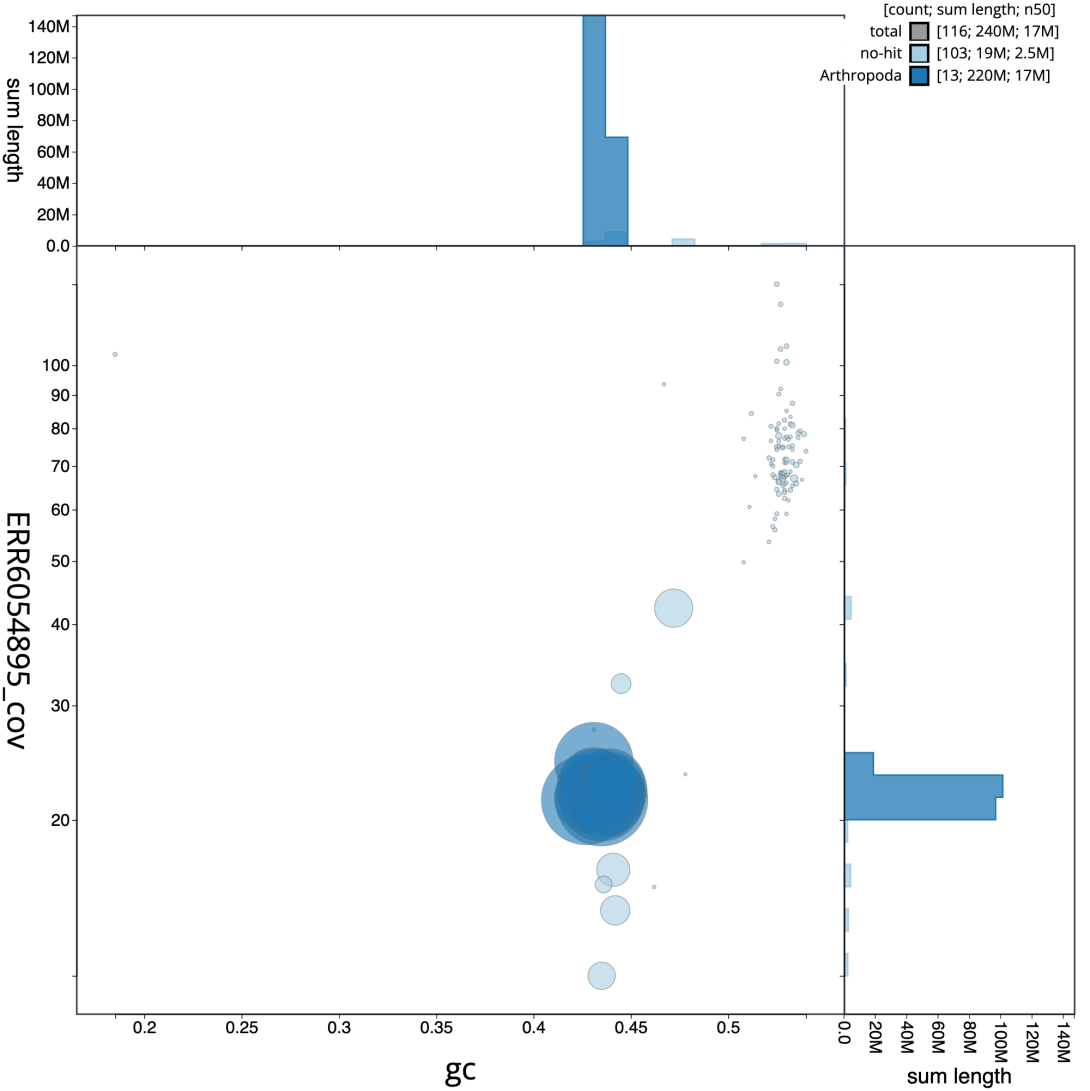
Genome assembly of *Ectemnius lituratus*, iyEctLitu1.2: BlobToolKit GC-coverage plot. Scaffolds are coloured by phylum. Circles are sized in proportion to scaffold length. Histograms show the distribution of scaffold length sum along each axis. An interactive version of this figure is available at https://blobtoolkit.genomehubs.org/view/Ectemnius%20lituratus/dataset/CAJVAQ02.1/blob.

**Figure 4.**
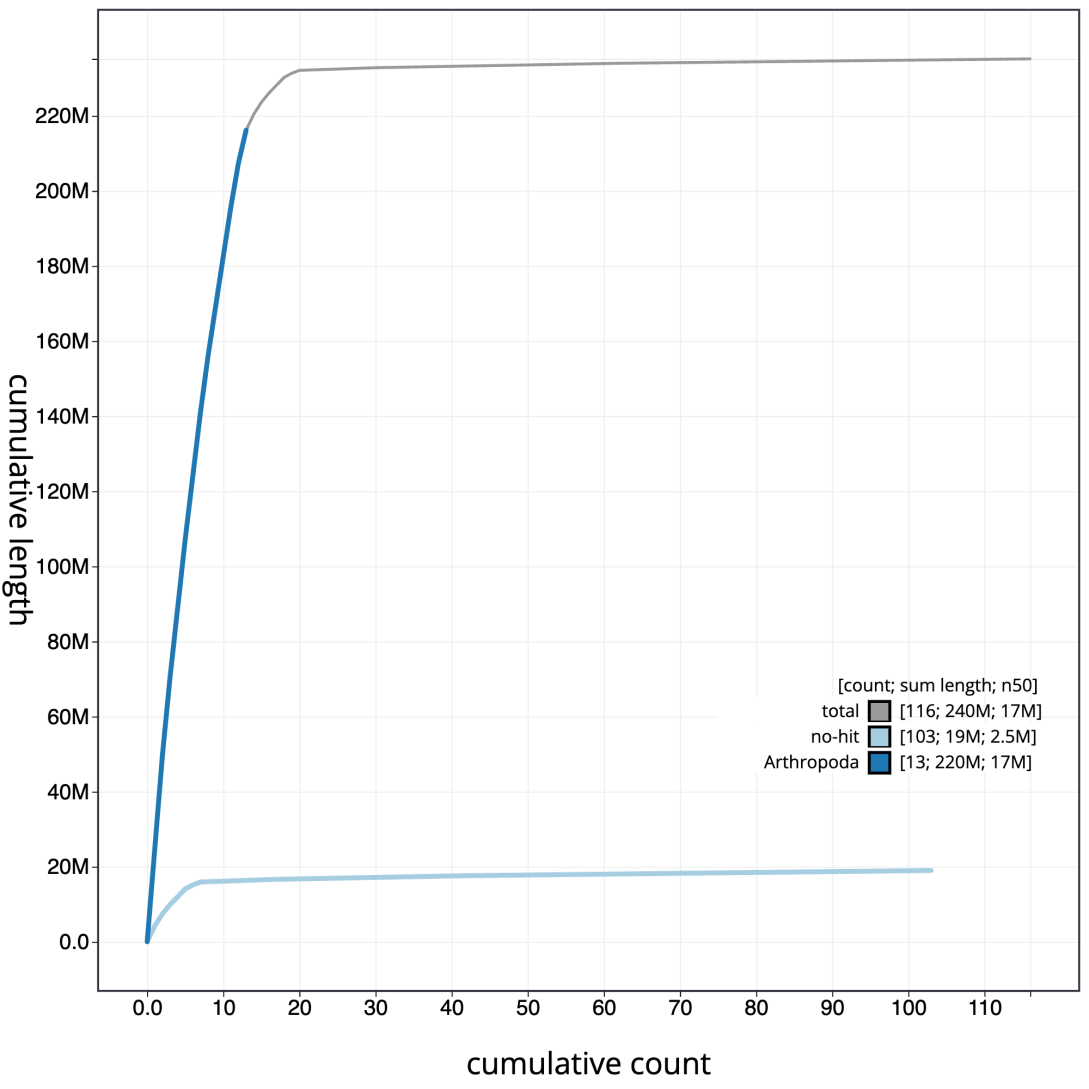
Genome assembly of *Ectemnius lituratus*, iyEctLitu1.2: BlobToolKit cumulative sequence plot. The grey line shows cumulative length for all scaffolds. Coloured lines show cumulative lengths of scaffolds assigned to each phylum using the buscogenes taxrule. An interactive version of this figure is available at https://blobtoolkit.genomehubs.org/view/Ectemnius%20lituratus/dataset/CAJVAQ02.1/cumulative.

**Figure 5.**
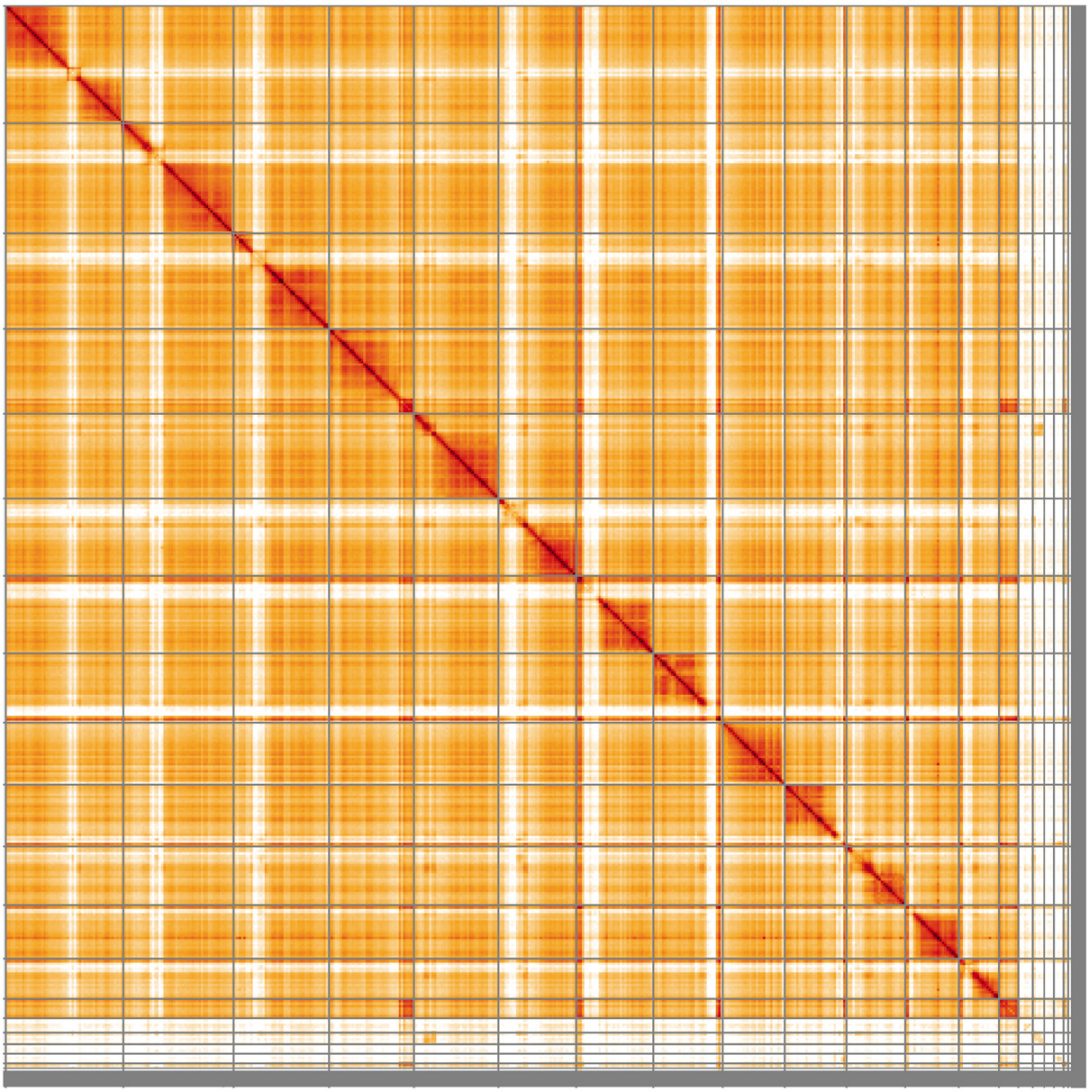
Genome assembly of *Ectemnius lituratus*, iyEctLitu1.2: Hi-C contact map of the iyEctLitu1.2 assembly, visualised using HiGlass. Chromosomes are shown in order of size from left to right and top to bottom. An interactive version of this figure may be viewed at https://genome-note-higlass.tol.sanger.ac.uk/l/?d=Ilu5QGtMSKO2rcgt0HMfYg.

The final assembly has a total length of 235.1 Mb in 115 sequence scaffolds with a scaffold N50 of 16.9 Mb ( Table 1). The snailplot in Figure 2 provides a summary of the assembly statistics, while the distribution of assembly scaffolds on GC proportion and coverage is shown in Figure 3. The cumulative assembly plot in Figure 4 shows curves for subsets of scaffolds assigned to different phyla. Most (93.16%) of the assembly sequence was assigned to 13 chromosomal-level scaffolds. Chromosome-scale scaffolds confirmed by the Hi-C data are named in order of size ( Figure 5; Table 2). While not fully phased, the assembly deposited is of one haplotype. Contigs corresponding to the second haplotype have also been deposited. The mitochondrial genome was also assembled and can be found as a contig within the multifasta file of the genome submission.

**Table 1.** Genome data for *Ectemnius lituratus*, iyEctLitu1.2.

Project accession data		
Assembly identifier	iyEctLitu1.2	
Assembly release date	2022-02-09	
Species	*Ectemnius lituratus*	
Specimen	iyEctLitu1	
NCBI taxonomy ID	2495015	
BioProject	PRJEB45182	
BioSample ID	SAMEA7520491	
Isolate information	iyEctLitu1	
Assembly metrics *		*Benchmark*
Consensus quality (QV)	50.5	*≥ 50*
*k*-mer completeness	99.99%	*≥ 95%*
BUSCO **	C:95.1%[S:95.0%,D:0.1%],F:1.2%,M:3.7%,n:5,991	*C ≥ 95%*
Percentage of assembly mapped to chromosomes	93.16%	*≥ 95%*
Sex chromosomes	-	*localised homologous pairs*
Organelles	Mitochondrial genome assembled	*complete single alleles*
Raw data accessions		
PacificBiosciences SEQUEL II	ERR6560801	
10X Genomics Illumina	ERR6054896, ERR6054894, ERR6054895, ERR6054897	
Hi-C Illumina	ERR6054898, ERR6054899, ERR6054900	
PolyA RNA-Seq Illumina	ERR6054901	
Genome assembly		
Assembly accession	GCA_910593735.2	
*Accession of alternate haplotype*	GCA_910593685.1	
Span (Mb)	235.1	
Number of contigs	359	
Contig N50 length (Mb)	1.6	
Number of scaffolds	115	
Scaffold N50 length (Mb)	16.9	
Longest scaffold (Mb)	25.6	
Genome annotation		
Number of protein-coding genes	9,724	
Number of non-coding genes	1,423	
Number of gene transcripts	17,960	

* Assembly metric benchmarks are adapted from column VGP-2020 of “Table 1: Proposed standards and metrics for defining genome assembly quality” from ( Rhie *et al.*, 2021). ** BUSCO scores based on the hymenoptera_odb10 BUSCO set using v5.3.2. C = complete [S = single copy, D = duplicated], F = fragmented, M = missing, n = number of orthologues in comparison. A full set of BUSCO scores is available at https://blobtoolkit.genomehubs.org/view/Ectemnius%20lituratus/dataset/CAJVAQ02.1/busco.

**Table 2.** Chromosomal pseudomolecules in the genome assembly of *Ectemnius lituratus*, iyEctLitu1.

INSDC accession	Chromosome	Length (Mb)	GC%
OU343033.1	1	25.57	43.5
OU343034.1	2	24.01	42.5
OU343035.1	3	20.75	43.5
OU343036.1	4	18.52	43.0
OU343037.1	5	18.38	43.0
OU343038.1	6	16.91	44.0
OU343039.1	7	16.85	44.0
OU343040.1	8	15.08	44.0
OU343041.1	9	13.52	43.0
OU343042.1	10	13.49	43.0
OU343043.1	11	12.71	43.5
OU343044.1	12	11.72	44.0
OU343045.1	13	8.66	44.0
OU343046.1	MT	0.03	18.5

The estimated Quality Value (QV) of the final assembly is 50.5 with *k*-mer completeness of 99.99%, and the assembly has a BUSCO v5.3.2 completeness of 95.1% (single = 95.0%, duplicated = 0.1%), using the hymenoptera_odb10 reference set ( *n* = 5,911.

Metadata for specimens, barcode results, spectra estimates, sequencing runs, contaminants and pre-curation assembly statistics are given at https://links.tol.sanger.ac.uk/species/2495015.

## Genome annotation report

The *Ectemnius lituratus* genome assembly (GCA_910593735.2) was annotated using the Ensembl rapid annotation pipeline ( Table 1; https://rapid.ensembl.org/Ectemnius_lituratus_GCA_910593735.2/Info/Index). The resulting annotation includes 17,960 transcribed mRNAs from 9,724 protein-coding genes.

## Methods

### Sample acquisition and nucleic acid extraction

The specimen used for genome sequencing and Hi-C data was a female *Ectemnius lituratus* (specimen ID Ox000161, ToLID iyEctLitu1), which was netted in Wytham Woods, Oxfordshire (biological vice-county Berkshire), UK (latitude 51.77, longitude –1.34) on 2019-08-07. The specimen used for RNA sequencing, also a female (specimen ID Ox000178, ToLID iyEctLitu2) was collected in the same location on 2019-08-13. The specimens were collected and identified by Liam Crowley (University of Oxford) and preserved on dry ice.

DNA was extracted at the Tree of Life laboratory, Wellcome Sanger Institute (WSI). The iyEctLitu1 sample was weighed and dissected on dry ice with tissue set aside for Hi-C sequencing. Head and thorax tissue was disrupted using a Nippi Powermasher fitted with a BioMasher pestle. High molecular weight (HMW) DNA was extracted using the Qiagen MagAttract HMW DNA extraction kit. Low molecular weight DNA was removed from a 20 ng aliquot of extracted DNA using the 0.8X AMpure XP purification kit prior to 10X Chromium sequencing; a minimum of 50 ng DNA was submitted for 10X sequencing. HMW DNA was sheared into an average fragment size of 12–20 kb in a Megaruptor 3 system with speed setting 30. Sheared DNA was purified by solid-phase reversible immobilisation using AMPure PB beads with a 1.8X ratio of beads to sample to remove the shorter fragments and concentrate the DNA sample. The concentration of the sheared and purified DNA was assessed using a Nanodrop spectrophotometer and Qubit Fluorometer and Qubit dsDNA High Sensitivity Assay kit. Fragment size distribution was evaluated by running the sample on the FemtoPulse system.

RNA was extracted from head and thorax tissue of iyEctLitu2 in the Tree of Life Laboratory at the WSI using TRIzol, according to the manufacturer’s instructions. RNA was then eluted in 50 μl RNAse-free water and its concentration assessed using a Nanodrop spectrophotometer and Qubit Fluorometer using the Qubit RNA Broad-Range (BR) Assay kit. Analysis of the integrity of the RNA was done using Agilent RNA 6000 Pico Kit and Eukaryotic Total RNA assay.

### Sequencing

Pacific Biosciences HiFi circular consensus and 10X Genomics read cloud DNA sequencing libraries were constructed according to the manufacturers’ instructions. Poly(A) RNA-Seq libraries were constructed using the NEB Ultra II RNA Library Prep kit. DNA and RNA sequencing was performed by the Scientific Operations core at the WSI on Pacific Biosciences SEQUEL II (HiFi), Illumina HiSeq 4000 (RNA-Seq) and HiSeq X Ten (10X) instruments. Hi-C data were also generated from abdomen tissue of iyEctLitu1 using the Arima2 kit and sequenced on the HiSeq X Ten instrument.

### Genome assembly, curation and evaluation

Assembly was carried out with Hifiasm ( Cheng *et al.*, 2021) and haplotypic duplication was identified and removed with purge_dups ( Guan *et al.*, 2020). One round of polishing was performed by aligning 10X Genomics read data to the assembly with Long Ranger ALIGN, calling variants with FreeBayes ( Garrison & Marth, 2012). The assembly was then scaffolded with Hi-C data ( Rao *et al.*, 2014) using SALSA2 ( Ghurye *et al.*, 2019). The assembly was checked for contamination and corrected using the gEVAL system ( Chow *et al.*, 2016) as described previously ( Howe *et al.*, 2021). Manual curation was performed using gEVAL, HiGlass ( Kerpedjiev *et al.*, 2018) and Pretext ( Harry, 2022). The mitochondrial genome was assembled using MitoHiFi ( Uliano-Silva *et al.*, 2023), which runs MitoFinder ( Allio *et al.*, 2020) or MITOS ( Bernt *et al.*, 2013) and uses these annotations to select the final mitochondrial contig and to ensure the general quality of the sequence.

A Hi-C map for the final assembly was produced using bwa-mem2 ( Vasimuddin *et al.*, 2019) in the Cooler file format ( Abdennur & Mirny, 2020). To assess the assembly metrics, the *k*-mer completeness and QV consensus quality values were calculated in Merqury ( Rhie *et al.*, 2020). This work was done using Nextflow ( Di Tommaso *et al.*, 2017) DSL2 pipelines “sanger-tol/readmapping” ( Surana *et al.*, 2023a) and “sanger-tol/genomenote” ( Surana *et al.*, 2023b). The genome was analysed within the BlobToolKit environment ( Challis *et al.*, 2020) and BUSCO scores ( Manni *et al.*, 2021; Simão *et al.*, 2015) were calculated.

Table 3 contains a list of relevant software tool versions and sources.

**Table 3.** Software tools: versions and sources.

Software tool	Version	Source
BlobToolKit	4.1.7	https://github.com/blobtoolkit/blobtoolkit
BUSCO	5.3.2	https://gitlab.com/ezlab/busco
FreeBayes	1.3.1-17-gaa2ace8	https://github.com/freebayes/freebayes
gEVAL	N/A	https://geval.org.uk/
Hifiasm	0.15	https://github.com/chhylp123/hifiasm
HiGlass	1.11.6	https://github.com/higlass/higlass
Long Ranger ALIGN	2.2.2	https://support.10xgenomics.com/genome-exome/ software/pipelines/latest/advanced/other-pipelines
Merqury	MerquryFK	https://github.com/thegenemyers/MERQURY.FK
MitoHiFi	2	https://github.com/marcelauliano/MitoHiFi
PretextView	0.2	https://github.com/wtsi-hpag/PretextView
purge_dups	1.2.3	https://github.com/dfguan/purge_dups
SALSA	2.2	https://github.com/salsa-rs/salsa
sanger-tol/genomenote	v1.0	https://github.com/sanger-tol/genomenote
sanger-tol/readmapping	1.1.0	https://github.com/sanger-tol/readmapping/tree/1.1.0

### Genome annotation

The Ensembl gene annotation system ( Aken *et al.*, 2016) was used to generate annotation for the *Ectemnius lituratus* assembly (GCA_910593735.2). Annotation was created primarily through alignment of transcriptomic data to the genome, with gap filling via protein-to-genome alignments of a select set of proteins from UniProt ( UniProt Consortium, 2019).

### Wellcome Sanger Institute – Legal and Governance

The materials that have contributed to this genome note have been supplied by a Darwin Tree of Life Partner. The submission of materials by a Darwin Tree of Life Partner is subject to the **‘Darwin Tree of Life Project Sampling Code of Practice’**, which can be found in full on the Darwin Tree of Life website here. By agreeing with and signing up to the Sampling Code of Practice, the Darwin Tree of Life Partner agrees they will meet the legal and ethical requirements and standards set out within this document in respect of all samples acquired for, and supplied to, the Darwin Tree of Life Project.

Further, the Wellcome Sanger Institute employs a process whereby due diligence is carried out proportionate to the nature of the materials themselves, and the circumstances under which they have been/are to be collected and provided for use. The purpose of this is to address and mitigate any potential legal and/or ethical implications of receipt and use of the materials as part of the research project, and to ensure that in doing so we align with best practice wherever possible. The overarching areas of consideration are:

•   Ethical review of provenance and sourcing of the material

•   Legality of collection, transfer and use (national and international)

Each transfer of samples is further undertaken according to a Research Collaboration Agreement or Material Transfer Agreement entered into by the Darwin Tree of Life Partner, Genome Research Limited (operating as the Wellcome Sanger Institute), and in some circumstances other Darwin Tree of Life collaborators.

## Data Availability

European Nucleotide Archive: *Ectemnius lituratus*. Accession number PRJEB45182; https://identifiers.org/ena.embl/PRJEB45182 ( Wellcome Sanger Institute, 2021). The genome sequence is released openly for reuse. The *Ectemnius lituratus* genome sequencing initiative is part of the Darwin Tree of Life (DToL) project. All raw sequence data and the assembly have been deposited in INSDC databases. Raw data and assembly accession identifiers are reported in Table 1.

## Author information

Members of the University of Oxford and Wytham Woods Genome Acquisition Lab are listed here: https://doi.org/10.5281/zenodo.7125292.

Members of the Darwin Tree of Life Barcoding collective are listed here: https://doi.org/10.5281/zenodo.4893703.

Members of the Wellcome Sanger Institute Tree of Life programme are listed here: https://doi.org/10.5281/zenodo.4783585.

Members of Wellcome Sanger Institute Scientific Operations: DNA Pipelines collective are listed here: https://doi.org/10.5281/zenodo.4790455.

Members of the Wellcome Sanger Institute Tree of Life Core Informatics Team are listed here:

Members of the Tree of Life Core Informatics collective are listed here: https://doi.org/10.5281/zenodo.5013541.

Members of the Darwin Tree of Life Consortium are listed here: https://doi.org/10.5281/zenodo.4783558.
