# Supplementary material for: The genome sequence of a digger wasp, Ectemnius lituratus (Panzer,1805)
Source: Wellcome Open Res. 2023 Dec 1;8:552. [Version 1] doi: 10.12688/wellcomeopenres.20337.1 (PMC11345589; doi:10.12688/wellcomeopenres.20337.1)
Supplement: Peer review history

# Reviewer report: Reviewer response for version 1

**DOI:** 10.21956/wellcomeopenres.22520.r80914

**Published:** 2024-08-25

**Authors:** Natalia de Souza Araujo

**Affiliations:** 1 Universite Libre de Bruxelles, Brussels, Brussels, Belgium

The authors report the sequencing and assembly of the mitochondrial and nuclear genomes of the digger wasp *Ectemnius lituratus*. The nuclear genome assembly span is ~235Mb, most of which occurs within 13 chromosomal scaffolds and contains 9,724 annotated protein code genes and 1,423 non-coding genes. The mitochondrial genome reported has a size of ~29kb.

 The authors give a brief introduction of the species biology, describe the sampling site, and have deposited the data in public sequencing repositories. The extraction and assembly methods are well established within the Darwin Tree of Life pipeline. In conclusion, this is a straightforward assembly report.

**Some minor suggestions are:**

1. In the Methods, *Genome assembly, curation and evaluation* section, the authors say “The mitochondrial genome was assembled using MitoHiFi…, which runs MitoFinder or MITOS…”. Please specify which one was used for the annotation in this specific assembly.
2. The mitochondrial genome annotation is not described in the report, and it is not clear if it contained or not all the expected genes not if the A+T region was recovered.
3. Figure 5, it would be nice to have the scaffolds delimitation in the plot, it is not clear what in the map indicates the chromosomes delimitations.

Are sufficient details of methods and materials provided to allow replication by others?

Yes

Is the rationale for creating the dataset(s) clearly described?

Yes

Are the datasets clearly presented in a useable and accessible format?

Yes

Are the protocols appropriate and is the work technically sound?

Yes

Reviewer Expertise:

Insect genomics and evolution

I confirm that I have read this submission and believe that I have an appropriate level of expertise to confirm that it is of an acceptable scientific standard.

# Reviewer report: Reviewer response for version 1

**DOI:** 10.21956/wellcomeopenres.22520.r77330

**Published:** 2024-03-27

**Authors:** Jonathan Berenguer Uhuad Koch

**Affiliations:** 1 USDA-ARS Pollinating Insect Research Unit, Maryland, USA

The authors present a high quality genome assembly of the digger wasp, Ectemnius lituratus (Panzer, 1805). The genome assembly was achieved with HiFi, 10x, and HiC sequencing. Annotation of the genome was achieved through sequencing RNA for annotation with Ensembl pipeline. Then genome assembly is 235.1 Mb and scaffolded into 13 chromosomal pseudo molecules. 

 The data note is well written and to the point. My only suggestion is as follows: What taxonomic resource was used as the authority to confirm species identity? Report either taxonomic key or original species description.

Are sufficient details of methods and materials provided to allow replication by others?

Yes

Is the rationale for creating the dataset(s) clearly described?

Yes

Are the datasets clearly presented in a useable and accessible format?

Yes

Are the protocols appropriate and is the work technically sound?

Yes

Reviewer Expertise:

population genetics, entomology, genomics

I confirm that I have read this submission and believe that I have an appropriate level of expertise to confirm that it is of an acceptable scientific standard.
